# Emergency and acute care management of traumatic spinal cord injury: a survey of current practice among senior clinicians across Australia

**DOI:** 10.1186/s12873-018-0207-0

**Published:** 2018-12-20

**Authors:** Lisa N. Sharwood, Shelly Dhaliwal, Jonathon Ball, Brian Burns, Oliver Flower, Anthony Joseph, Ralph Stanford, James Middleton

**Affiliations:** 1 John Walsh Centre for Rehabilitation Research, Kolling Institute, Northern Clinical School, Sydney Medical School, University of Sydney, Reserve Road, St Leonards, NSW 2065 Australia; 2 Department of Neurosurgery, Royal North Shore Hospital, Reserve Road, St Leonards, NSW 2065 Australia; 3 Greater Sydney Area Helicopter Emergency Medical Service, Bankstown, NSW 2200 Australia; 4 Intensive Care Department, Royal North Shore Hospital, Reserve Road, St Leonards, NSW 2065 Australia; 5 Department of Trauma, Royal North Shore Hospital, Reserve Road, St Leonards, NSW 2065 Australia; 6 Department of Orthopaedics, Prince of Wales Private Hospital, Barker Street, Randwick, NSW 2031 Australia

## Abstract

**Background:**

To describe pre-hospital, emergency department and acute care assessment and management practices of senior clinicians for patients with acute traumatic spinal cord injury (TSCI) across Australia; and to describe clinical practice variation.

**Methods:**

We used a descriptive, cross-sectional study design to survey senior clinicians (greater than 10 years practice in this field) caring for patients with acute TSCI. The assessment, management and referral practices of prehospital, emergency department/trauma and surgical expert clinicians, across prehospital, early hospital care, diagnostic imaging and haemodynamic management were surveyed.

**Results:**

We invited 95 eligible senior clinicians; the response rate was 75%. Survey findings demonstrated overall lack of awareness or consistent use of evidence based published guidelines; many clinicians following ‘locally written’ or ‘no particular’ guideline. Practitioners were conflicted across multiple areas including patient assessment and diagnosis, treatment and transport decisions. Reported spinal immobilisation practices differed substantially, as did target setting for blood pressure; the majority of clinicians actively monitored risk of respiratory deterioration. Specialist care consult and specialist service bed availability was reported as problematic by more than one third of clinicians.

**Conclusions:**

Unwarranted clinical practice variation is known to contribute to different health outcomes for patients with similar etiologies. Clinical practice guidelines offer evidence based, best practice standards, however are only effective if adopted throughout the healthcare system. Wide variability in acute care practices, pathways and timing to specialist centres for TSCI was evidenced by this survey despite seniority among clinicians. This devastating injury requires prompt, consistent, evidence based care from the moment of first responder. Improved outcomes for patients with TSCI would be more likely with standardised care across pre-hospital, emergency and acute care phases of care.

**Keywords:**

Spinal Cord Injuries, Multiple Trauma, Practice Guideline, Treatment Outcome, Surveys and Questionnaires, Expert Testimony

## Background

The pathway that an injured patient follows from the incident location to definitive care can be variable and cover large geographical distances in Australia. Patients with traumatic spinal cord injury (TSCI) require skilled assessment, appropriate management and timely access to specialist treatment. This is most vital in the immediate post injury phase, where failure to adhere to evidence based guidelines and standards poses serious risk of secondary neurological deterioration [1]. Inconsistencies in acute care protocols and health service pathways to access specialist care have been identified in this injury group, prompting calls for nationally consistent care standards [2]. The risk of secondary complications in patients with TSCI is increased for those who do not obtain acute care in a specialist spinal cord injury unit (SCIU) within 24 h from the time of injury [3, 4]. Parent et al. have also systematically reviewed the impact of SCIU care on TSCI complications and mortality, concluding that it reduced lengths of stay, mortality risk and the number and severity of complications [5]. The extent to which these findings impact the pre-hospital, emergency and surgical clinical practice and transfer decisions of senior clinicians in Australia is unknown.

Internationally, clinical practice variation and disagreement surrounding best practice has been reported by systematic review [6]. Unwarranted clinical practice variation can result from differences in staff capabilities and local protocols of care, and can lead to different health outcomes for patients with similar etiologies [7]. A recent survey of all neurosurgical units in the United Kingdom reported wide variability among neurosurgeons, neuro-anaesthetists and intensivists in their treatment of acute TSCI, including intensive medical management to improve neurological outcome [8]. Information regarding clinical practice variation is important to document in order to determine the efficiency and effectiveness of a health care service. From this baseline, quality improvements can be benchmarked.

The practice of spinal immobilisation for suspected TSCI in the pre-hospital phase has been the subject of contention in recent years. Ahn et al. [6] described significant variability in practice during pre-hospital and inter-hospital transfer phases. Following systematic review and a Delphi process, they recommended that the emergency medical personnel should be trained to clear patients of cervical injury or immobilise patients suspected of a cervical spinal injury in a pre-hospital setting. They specified immobilisation to include a cervical collar, head immobilization, and a spinal board. Connor et al’s consensus statement [9] however, concluded the long spinal board is an extrication device solely, and ‘manual in-line stabilisation’ as an ‘appropriate substitute’ for a cervical collar; likely better for patients with airway compromise or concomitant head injury. In Australia, the Queensland Ambulance Service has followed Bergen (Norway), the Netherlands and Northern California (USA) where the routine use of rigid c-spine extrication collars has been abandoned in pre-hospital settings. The NEXUS criteria is used to clear suspected cervical spine injury; where not possible to do so, a soft foam collar is applied for suspected cervical TSCI. Also in the early hospital setting, controversy surrounds both the appropriateness of spinal immobilisation, and the correct choice of methods or devices [10, 11].

Available levels of evidence influence consensus and practice in clinical settings. For example, the recommendation to maintain mean arterial pressure above 85-90 mmHg for up to seven days post-TSCI [12], is based on Level II evidence only. Adhering to this guideline often requires intravenous vasopressors and invasive monitoring (requiring Intensive Care Unit admission). A systematic review of animal studies has demonstrated both favorable and unfavorable findings from this practice, concluding the evidence as completely inconclusive [13].

Evidence informs the development of clinical practice guidelines (CPG); which aim to standardise and improve health care quality, reduce the likelihood of ineffective interventions and maximise best possible outcomes for the patient [14]. They are, however, only effective if used consistently in clinical practice and decision-making. In the acute care of patients with TSCI, there are some CPGs in existence; published by the American Association of Neurological Surgeons (AANS) [15], the Paralyzed Veterans of America (PVA) [16], and most recently by an international group led by Fehlings in Canada [17, 18]. The degree to which these internationally published guidelines are followed, nor the extent of variability in the acute care of TSCI across Australia has not been previously documented. Without such information, it is not possible to understand drivers of any gap between evidence and practice, nor to undertake any benchmarking to improve and standardise the care for patients with TSCI and ensure best possible outcomes with reduced long-term costs. This study aimed therefore to examine self-reported practice, including subscription or adherence to particular clinical practice protocols in the early acute care of patients with TSCI, to provide an indication of variability among senior clinicians across Australia.

## Methods

We used a descriptive cross-sectional study design to survey a select group of senior clinicians about their assessment and management of patients with acute TSCI. A questionnaire was developed by a steering committee that consisted of clinicians and academic researchers experienced in the acute care of patients with TSCI. The steering committee (with the assistance of the NSW Health Institute of Trauma and Injury Management) compiled a list of 95 clinicians nation-wide who were deemed ‘experts’ in their field. Eligible clinicians were those with 10 years or more experience providing acute care for patients with TSCI; practicing across rural, metropolitan or greater metropolitan areas. They included pre-hospital paramedics and retrieval doctors, trauma physicians, critical care nurses, orthopedic surgeons and neurosurgeons, and intensive care specialists. The RedCap [19] web-based application was utilised to build the online survey, then collect and analyse data. The survey was sent as an embedded link via a personalized email. It was then self-administered; all questions were open to all respondents, however, clinicians were encouraged to decline answering questions that were not applicable to their specific expertise or area of practice. Most questions had multiple response choices that were identified from the literature, however, all questions had free text boxes for additional answers.

## Results

Completed surveys were submitted by 71of the 95 eligible senior clinicians contacted (response rate 74.7%), written consent was obtained. Over two-thirds of respondents (69%, *n* = 49) had more than 15 years’ experience in TSCI acute care; the remainder had between 10 and 15 years’ experience. Professional groups comprised the pre-hospital clinicians (29%, *n* = 22); emergency/trauma department doctors (28%, *n* = 21); trauma specialist nurses (21%, *n* = 15); intensivists (5%, *n* = 4); orthopedic surgeons (8%, *n* = 6); neurosurgeons (4%, *n* = 3) and trauma surgeons (3%, *n* = 2). Most clinicians were practicing in a Metropolitan area (67.1%, *n* = 47); 18.6% (*n* = 13) in a greater metropolitan setting; and 14.3% (*n* = 10) in a rural setting. Findings are described below for the four clinical areas explored in this survey of current practice.

### Pre-hospital care

Slightly more than 10% of respondents (12.3%, *n* = 8) reported using the AANS guidelines for prehospital cervical spinal immobilisation and prehospital transportation to direct their care of the patient with acute TSCI. The majority of respondents (61.5%, *n* = 40) indicated they used ‘locally written’ guidelines (Table 1). Factors most likely to impede direct transfer of patients from the scene of injury to the SCIU included comorbid injury (38.7%, *n* = 24), policy or protocols not permitting this practice (38.7%, *n* = 24) and geographical distance (32.2%, *n* = 20). Clinicians reported to a lesser extent the lack of available aeromedical transport (19.3%, *n* = 12) or SCIU bed (19.3%, *n* = 12). Lack of access to SCIU beds was additionally noted to relate to the absence of a non-refusal policy as frustrating this problem (i.e. that the SCIU must accept an acute TSCI patient, regardless of bed capacity). Around one-third (36%, *n* = 23) of respondents thought that the SCIU in their region had such a policy in place, and adhered to it (Table 1).

**Table 1:** Pre Hospital Care Questions and Responses

Survey Question (*n* = respondents per question)	Response – [n (%)]
1. What protocols guide the care of the patient with acute TSCI in the early hospital phase in your region of practice? (*n* = 65)	• Paralysed Veterans of America guides (PVA) 2008 [1 (1.5)] • American Association of Neurological Surgeons/ Congress of Neurological Surgeons (AANS/CNS) 2013 [8 (12.3)] • American College of Surgeons – Advanced Trauma Life Support (ATLS) [20 (30.8)] • Locally written [40 (61.5)] • Other [8 (12.3)] • None [5 (7.7)]
2. Do you believe neurological assessment in the field is able to correctly identify all cases of TSCI (sensitivity) as well as exclude people without? (*n* = 66)	• Yes [20 (30.3)] • No [40 (60.6)] • Not sure [6 (9.1)]
3. In your region of practice, can a pre-hospital clinician make the decision to transport the patient with an apparent isolated TSCI directly to a dedicated SCI service? (*n* = 65)	• Yes [37 (56.9)] • No [20 (30.8)] • Not sure [8 (12.3)]
4. In your view, should all patients with a suspected TSCI (apparent paralysis and/or numbness or sufficient doubt because of altered mental state or major injury) receive spinal immobilization at the scene of injury? (*n* = 66)	• Yes [64 (97)] • No [2 (3)]
5. In your view should all patients with a potential TSCI (due to mechanism, spinal pain or sufficient doubt because of altered mental state or major injury), but no apparent paralysis or numbness receive spinal immobilization at the scene of injury? (*n* = 66)	• Yes [50 (75.8) • No [16 (24.2)]
6. What devices (or combination of devices) would the clinician use for extrication? (NB: participants could choose multiple responses) (*n* = 66)	• Rigid extrication collar [51 (77.3)] • Backboard with straps [47 (71.2)] • Semi-rigid cervical collar [14 (21.2)] • Soft foam collar [3 (4.5)] • Sandbags [30 (45.5)] • Other [18 (27.3)] (free responses included the ‘Kendrick extraction device, ‘NEANN Immobilisation and Extraction Jacket’ (NIEJ); scoop for extrication; vacuum mattress and backboard with no straps)
7. What devices would you use for spinal immobilization for transport? (NB: participants could choose multiple responses) (*n* = 66)	• Rigid extrication collar [51 (77.3)] • Backboard with straps [27 (40.9)] • Semi-rigid cervical collar [15 (22.7)] • Soft foam collar [5 (7.6)] • Sandbags [39 (59.1)] • Other [12 (18.2)] (such as vacuum mattress, stretcher if transport > 60 min; stretcher harness and coop stretcher with straps)
8. In your region of practice, are patients with a TSCI transferred directly from the scene of injury to the SCIU in your state? (*n* = 66)	• Rarely [12 (18)] • Sometimes [33 (50)] • Most of the time [19 (28.8)] • Always [2 (3)]
9. In your region of practice, are patients with a TSCI transferred initially from the scene of injury to a major trauma service (without a co-located SCIU) in your state? (*n* = 65)	• Rarely [8 (12.3)] • Sometimes [14 (21.5)] • Most of the time [33 (50.8)] • Always [10 (15.4)]
10. In your region of practice, is it always achievable to contact the SCIU within 2 h of the patient injury and achieve transfer within 24 h? (*n* = 60)	• Yes [38 (63.3)] • No [22 (36.7)]
11. In your region of practice, does the SCIU have, and adhere to a ‘non-refusal’ policy? (*n* = 64)	• Yes [23 (35.9)] • No [19 (29.7)] • Don’t know [22 (34.4)]
12. In your region of practice, is airway intubation ever performed in the field in the setting of TSCI? (*n* = 64)	• Yes [49 (76.6)] • No [2 (3.1)] • Don’t know [13 (20.3)]
13. If not currently practiced, in the clinician’s view, is there a role for airway intubation in the field in appropriate cases of patients with TSCI? (*n* = 56)	• Yes [51 (91.1)] • No [5 (8.9)]

Some free-text comments suggested that the practice of direct transfer to a specialist SCIU may in some instances be influenced by clinician beliefs and attitudes, such as this comment made by a Paramedic with more than 15 years of experience:

“Guidelines provided to us state no proven benefit in transporting patients with suspected or proven spinal cord injuries by helicopter (transport mode to nearest specialist spinal centre). They further state it is extremely rare for a patient with TSCI to have improved neurological outcome by urgent surgery at a spinal injuries referral centre, as damage is overwhelmingly done at time of injury.” (Anonymous)

### Early hospital care

Regarding organisationally proscribed guidelines to direct their early hospital care of patients with acute TSCI, the majority of clinicians (45.1%, *n* = 32) were unable to name a particular protocol guiding their care. Around 30% (*n* = 22) reported using a particular trauma protocol with a minority (5.6%, *n* = 4) recording use of a statewide spinal cord service protocol. Some respondents wrote that they ‘did not have’ such a protocol in their state (Table 2).

**Table 2:** Early Hospital Care Questions and Responses

Survey Question (*n* = respondents per question)	Response – [n (%)]
1. What organisationally proscribed guidelines guide your early hospital care of patients with acute TSCI? (*n* = 71)	• Unsure [20 (28.1) • No particular guideline [12 (16.9)] • Trauma Protocols including Early Management of Severe Trauma, Advanced Trauma Life Support®, state-based Major Trauma guidelines, other Trauma Protocol [22 (30.5)] • Locally written [11 (15.5)] • Statewide Spinal Cord Service Protocol [4 (5.6)] • AANS recommendations [2 (2.8)]
2. In your current practice, do you aim to remove patients from a backboard within 15 min from their arrival? (*n* = 61)	• Yes [44 (72.1)] • No time frame [5 (8.2)] • Not sure [4 (6.6)] • Other [8 (13.1) (“never use backboard for transport”, “remove backboard as soon as patient on stretcher with spinal rated mattress”)
3. What methods do you use to protect the cervical spine? (*n* = 60) (participants could choose multiple responses)	• Leave rigid collar in situ [25 (41.7)] • Replace rigid collar with a semi rigid collar (Philadelphia/Aspen/similar) [35 (58.3)] • Sand-bags and tapes [20 (33.3)] • Other [12 (20)] (e.g. sandbags with no tapes; spine splints, vacuum mattress; manual in-line stabilisation, stretcher harness and handling such as log roll)
4. In which patients do you utilize the log-roll manoeuvre? (*n* = 60)	• All major trauma (defined by mechanism or physiological parameters) [49 (79)] • Only those with suspected spinal column injury (mechanism and/or spinal pain, presence of confounding factors) [17 (27.4)] • Only those with neurological deficit [2 (3.2)]
5. If you log-roll, how many staff members are called upon to move, turn the patient needing spinal immobilization? (*n* = 62)	• 2 [2 (3.2)] • 3 [10 (16.1)] • 4 [43 (69.3)] • 5 [5 (14.5)] • 6 [2 (3.2)]
6. In your practice how frequently do you perform pressure area care with skin inspection? (*n* = 57)	• Every hour [7 (12.3)] • Every 2 h [29 (50.9)] • Every 3 h [3 (5.3)] • Every 4 h [1 (1.8)] • Once per shift [2 (3.5)] • Other [15 (26.3)] (most reported following instructions from spinal physicians)
7. Do you have specialised beds available within 2 h of a patient with confirmed TSCI being admitted to your service? (*n* = 57)	• Yes [19 (33.3)] • No [28 (49.1)] • Not sure [10 (17.5)]
8. What indicators of respiratory failure do you measure in known cervical spinal cord injury? (*n* = 55)	• Vital capacity [4 (7.3)] • Arterial blood gases [14 (25.5)] • Both of the above [22 (52.7)] • Other [15 (27.3)] (included pulse oximetry, tidal volume, and respiratory rate)
9. What are your indications (any or all) to intubate patients with a cervical spinal cord injury? (participants could choose multiple responses) (*n* = 58)	• Clinical evidence of respiratory distress [51 (87.9) • Poor or deteriorating vital capacity/ABGs [44 (75.9)] • All cases prior to air transport [5 (8.6)]
10. Is high dose methylprednisolone used in your area of practice to treat TSCI? (*n* = 55)	• Yes [6 (10.9)] • No [49 (89.1)]

Practices for cervical spine protection varied greatly with differences reported in the use and types of cervical collars, staff numbers implicated for log-rolling of patients with suspected TSCI and frequencies of pressure area care. Clinicians used differing indicators of respiratory distress and the need to intubate a patient with TSCI in the early hospital phase (Table 2).

### Diagnostic imaging

Most clinicians had 24 h access to high quality CT (92.7%), however only three-quarters (76.4%) reported 24-h access to an MRI at their facility. Forty two percent (*n* = 19) of responding clinicians did not use MRI scans to influence their early decision-making and management of patients with acute TSCI. Others used MRI to diagnose ligamentous injury as part of TSCI (28.5%). Criteria used to guide clearance of any cord injury in the trauma patient varied between the Canadian C-spine rule (40%), NEXUS criteria (36%), both or other (24%) (Table 3).

**Table 3:** Diagnostic Imaging Questions and Responses

Survey Question (*n* = respondents per question)	Response – [n (%)]
11. Do you have high quality CT available 24 h? (*n* = 55)	• Yes [51 (92.7)] • No [4 (7.3)]
12. Do you have MRI available 24 h? (*n* = 55)	• Yes [42 (76.4)] • No [13 (23.6)]
13. What is the initial imaging technique you choose for patients with suspected spinal injury, with or without paralysis? (*n* = 46)	• 3-view spine x-ray series [4 (8.7)] • CT scanning [42 (91.3)]
14. What criteria do you use to ‘clear’ the spine on clinical grounds? (*n* = 50)	• Canadian C-spine rule [20 (40)] • NEXUS [18 (36)] • Other [12 (24%)] (referred to using both the NEXUS and Canadian C-Spine rules; the Dynamic Neck Exam; Alfred Hospital guidelines for C spine clearance)
15. Does MRI influence your early decision-making and management following acute SCI? (*n* = 46)	• Yes [28 (60.9)] • No [18 (39.1)]

### Haemodynamic management

For clinicians who aimed for a specific target blood pressure in patients with acute TSCI (61.4%, *n* = 35), reported mean arterial pressure (MAP) targets varied from 60 to 70 mmHg (34.3%, *N* = 12) to 80-90 mmHg (37.1%, *n* = 13) (Table 4). One third of clinicians did not attribute hypertension therapy following acute TSCI to improved neurological outcomes (33.3%, *n* = 19), however the majority considered concomitant penetrating trauma causing significant bleeding to contraindicate initiation of hypertensive therapy (82.2%, *n* = 37).

**Table 4:** Haemodynamic Management Questions and Responses

Survey Question	Response – n, %(n/n total respondents for that particular question)
16. Is there a target blood pressure for acute TSCI patients? (*n* = 57)	• Yes [35 (61.4%)] • No [22 (38.6%)]
17. If yes to above, what is the target for mean arterial blood pressure (mmHg) (*n* = 35)	• 60–70 mmHg [12 (34.3%)] • 71-80 mmHg [10 (28.5%)] • 81–90 mmHg [13 (37.1%)]
18. Target systolic blood pressure? (*n* = 17)	• 85–95 mmHg [6 (35.3%)] • 100–110 mmHg [7 (41.2%)] • 115–120 mmHg [4 (23.5%)]
19. Are patients kept in ICU for entire period of induced hypertension therapy? (*n* = 57)	• Yes [36 (63.2%)] • No [21 (36.8%)]
20. Are there any patient groups for which active BP management does not apply? (*n* = 41)	• Yes [11 (26.8%)] (patients with major haemorrhage requiring surgery; elderly with comorbidities; complete cord transection, high cervical injuries and life-threatening injuries including penetrating truncal injury with central pulses present) • No [30 (73.2%)]
21. Do you believe hypertension therapy following acute TSCI has an impact on neurological outcome? (n = 57)	• Yes [38 (66.7%)] • No [19 (33.3%)]
22. Do you aim to increase heart-rate in the bradycardia patient with TSCI in neurogenic shock? (*n* = 44)	• Yes [13 (29.5%)] (only if their heart rate was < 40 and MAP < 90; or if they were physiologically compromised, hypovolaemic or if perfusion remained inadequate despite fluid resuscitation) • No [31 (71.5%)]
23. If you have excluded hypovolemia, do you still aim to maintain a target blood pressure for treatment of TSCI? (*n* = 48)	• Yes [44 (91.7%)] • No [4 (8.3%)]
24. Does the presence of a TBI influence your management of blood pressure for TSCI in the same patient? (*n* = 46)	• Yes [26 (56.53%)] (depended on the TBI-specific targets such as the target Cerebral Perfusion Pressure, Intracranial Pressure). No [20 (43.5%)]
25. Do you think concomitant penetrating trauma causing significant bleeding is a contraindication to initiating hypertension therapy following acute TSCI? (*n* = 45)	• Yes [37 (82.2%)] • No [8 (17.8%)]

## Discussion

This comprehensive survey has canvassed current practices of a large sample (response rate 75%) of prehospital and acute care clinicians; all with extensive experience in the care of patients with acute TSCI. We have identified significant variability of practice across the prehospital and early hospital period for these patients; variation that is substantially greater than can reasonably be explained by the injury condition alone. Specifically, there are various published guidelines which make evidence based recommendations across these areas of clinical practice in patients with TSCI [12, 15, 16, 20, 21]. This survey finds that they are not well known in Australia, and the recommendations within, inconsistently applied in practice.

The contention regarding pre-hospital spinal immobilisation across Australia was evidenced by the differences in opinion as to whether patients with potential TSCI should receive immobilisation at the scene; one quarter of clinicians denying this was necessary where no apparent paralysis was evident (Table 1). The specific interpretation of these questions was not ascertained however; as such they make have considered using other methods of less rigid motion restriction. Approximately 60% of clinicians believed prehospital neurological assessment to have sufficient sensitivity and specificity to correctly identify TSCI in the field. This has obvious potential to impact treatment decisions and is in fact not evidence based. This is an area requiring prompt attention to enable safe and appropriate triage, management and transport decisions to be made in the field. Almost 60% of clinicians reported they would use sandbags for spinal immobilisation, yet this practice was not recommended following evidence review and published by the AANS in 2013 [21].

In the early hospital phase, pressure area care occurred every 1–2 h in over 60%, although specialised beds were only available in 33%. Providing care on a suitable mattress, depending on the patient’s condition and the stability of the fracture, is essential along with meticulous skin care and repositioning to provide pressure relief or turn every 2 h while maintaining spinal precautions. Monitoring with arterial blood gases occurred in around 80%, with pulse oximetry, vital capacity or other clinical assessments done in remainder. Appropriate consideration was given to need for intubation where there was evidence of respiratory distress, poor or deteriorating vital capacity. The importance of this practice recognises the potential risk of progressive ventilatory failure in patients with TSCI at or above the C6 level.

Timely contact with (< 2 h) and transfer to the SCIU within 24 h was reported as not always possible by around 40% of respondents, and 34.4% of respondents (*n* = 22) did not know if their service had a non-refusal policy in place. Given the increased of risk of mortality, secondary complications and longer length of stay clearly evidenced in patients who do not receive timely specialist care for TSCI [3–5]; these documented insufficiencies across healthcare services will likely impact patient outcomes. A nationally adopted best-practice standard on this will contribute towards preventing unnecessary delays in admission to a SCIU.

Twenty-four hour access to magnetic resonance imaging (MRI) was mostly available (76%), being reported to influence decision-making and clinical management in 61% of experienced clinicians. MRI can accurately detect potentially important radiological features involved in the spinal injury, such as ongoing cord compression, large disc herniation and ligamentous instability not evident on CT [22]. A recent systematic review [23] concluded that MRI may be a useful adjunct to assessment of baseline neurological status for prognostication*;* a resultant recommendation was that MRI be performed in adult patients with acute TSCI prior to surgical intervention to facilitate improved clinical decision-making, and improve prediction of neurologic outcome.

Reported targets set for blood pressure in the haemodynamic management of the patient with TSCI, varied between 60 and 70 mmHg (36%), 71-80 mmHg (25%) and > 80 mmHg (39%). Inconsistency in practice could be attributed to unavailable Level I evidence; findings here were very similar to results of a questionnaire conducted across neurosurgical units in the United Kingdom in 2012 [24]. Hopefully, the forthcoming findings from a currently recruiting non-inferiority trial comparing the avoidance of hypotension (MAP ≥65 mmHg) versus induced hypertension (MAP ≥85 mmHg) [25], will assist in clarifying this uncertainty.

The main limitations of this survey were the fact that despite seniority of practice, it is evident that not all clinicians were competent to respond to every question, where surgeons do not practice in the pre-hospital setting, nor Ambulance officers in the operating theatre. However, the natural selection of non-response to such context specific queries aimed at reducing the impact of this limitation, albeit also reducing the sample size. Also, having invited recommended clinicians from a nation-wide list of ‘experts’ in their field, this led to survey respondents representing five states in Australia yet we did not adjust our findings by state based differences in geography or trauma and SCIU availability. The reason for this was to derive a clear understanding of consistency despite state based variability, however the impact of location on response is unknown.

An obvious further limitation is the unknown result of this variability in practice of this experienced group of practitioners on the patients they were treating. There are many variables that come into play in the decision to treat a certain patient in a particular way, including comorbid injuries, for example. While the expertise of the practitioner is called upon to vary their practice in response to these individual patient variants, notwithstanding, consistency in practice is an overarching principal. Further study is needed to investigate the full impact of such variability on patient outcomes. It may be that clinical variability does not impact patient outcomes significantly. However, given the expert guidelines to which we refer, have been developed with thorough review of the best available evidence, we have deemed that these recommendations exist because they drive best practice for the best patient outcomes.

## Conclusions

The results of this survey have helped to identify areas of contention and uncertainty in the early clinical care of patients with TSCI. Benchmarking at least some of these areas to standardise practice to the best evidence available, in particular using internationally published CPGs where available, can help to ensure that patients consistently receive the highest quality of care. Setting a nationally standardised model of care that incorporates existing health services with specialist care for patients with TSCI will not only reduce secondary complications and rehabilitation barriers for patients at an individual level, but will also alleviate pressures on their carers and the healthcare system overall.

## Abbreviations

AANS: American Association of Neurological Surgeons
ATLS: Advanced Trauma Life Support
CPG: Clinical practice guidelines
MRI: Magnetic resonance imaging
PVA: Paralyzed veterans of America
SCIU: Spinal cord injury unit
TSCI: Traumatic spinal cord injury
